# Simulation-based Training Curriculum for the Management of Vaginal Cuff Dehiscence and Evisceration

**DOI:** 10.7759/cureus.6752

**Published:** 2020-01-23

**Authors:** Amelia D Chapman, Marla Bashour, Lauren Sagaria, David Gothard, Derek A Ballas

**Affiliations:** 1 Medicine, Ohio University Heritage College of Osteopathic Medicine, Warrensville Heights, USA; 2 Medical Education and Simulation, Northeast Ohio Medical University, Rootstown, USA; 3 Obstetrics and Gynecology, Summa Health System, Akron, USA; 4 Medicine, Summa Health System, Akron, USA

**Keywords:** vaginal cuff dehiscence, bowel evisceration, medical education, simulation, obstetrics and gynecology

## Abstract

Objective

Vaginal cuff dehiscence with evisceration (VCDE) is a serious, life-threatening complication of hysterectomy. Due to the high volume of hysterectomies performed in the U.S each year, it is likely that a practitioner will encounter VCDE during their career. Due to its infrequent occurrence, residents receive little exposure to it during training. Delayed diagnosis of VCDE can impede proper management and lead to severe, long-term complications or death. Our goal was to provide an opportunity for resident physicians to identify VCDE and practice performing a reduction of prolapsed bowel and vaginal cuff repair through hands-on simulation in hopes that the simulation would improve the residents’ confidence and knowledge in recognizing and managing future VCDE cases.

Methods

Obstetrics and Gynecology residents postgraduate year (PGY) 1-4 participated in this study (n=13). Before and after the simulated case, a knowledge test covering VCDE recognition and management and a confidence survey were given to the participants. A gynecologic mannequin was modified by placing simulated bowel into the abdominal cavity with a portion extending through a vaginal cuff and protruding from the vaginal introitus. For the simulation, a hemodynamically unstable patient presented with findings consistent with a VCDE. Once the decision to proceed to surgery was made, participants were transferred to a simulated operating room where they performed a reduction of prolapsed bowel and vaginal cuff closure either laparoscopically or vaginally on the mannequin. A debriefing session was held post-simulation to discuss management and thought processes, as well as reflect on their performance and discuss improvement strategies for future cases. Finally, the residents participated in a brief didactic lecture on education about the incidence, presentation, and management of VCDE.

Results

Analysis of the knowledge questionnaires showed the median score and interquartile range (IQR) pre- and post-simulation was 15(12-28) and 20(19-22) respectively, with a median score increase (and IQR) of 5(3.5-8.5) (p=0.001). The confidence score had pre- and post-simulation median scores (and IQRs) of 28(20-34.5) and 40(37.5-46) respectively, with a median score increase (and IQR) of 15(8-20.5) (p=0.001).

Conclusions

Our intervention improved residents’ knowledge and confidence in recognizing VCDE, identifying the need for surgical management, and performing a reduction of prolapsed bowel and vaginal cuff repair.

## Introduction

Hysterectomy is the second most common gynecologic procedure performed in the U.S for women of reproductive age [1]. With approximately 600,000 occurring annually, it is experienced by one in three women by the age of 60 [1,2]. Regardless of the mode of hysterectomy (vaginal, laparoscopic, robotic, or abdominal), all are associated with complications of infection, thromboembolism, genitourinary tract injury, and also a rare but serious complication of vaginal cuff dehiscence (VCD) [3]. VCD has been defined as partial or full-thickness separation of the anterior and posterior edges of the vaginal cuff created during a hysterectomy and can occur with or without bowel evisceration [4]. Proposed risk factors for dehiscence include vaginal atrophy, smoking, obesity, and pre-existing chronic conditions that increase intra-abdominal pressure [5].

Although VCD is rare, occurring in 0.14% to 4.1% of hysterectomies, it presents with serious, life-threatening complications such as bowel evisceration and necrosis [6,7]. Evisceration is typically of the distal ileum, but can include the omentum, appendix, and fallopian tubes, and is observed in 35%-65% of all VCD cases [4,6,8]. Evisceration of the distal ileum can lead to bowel necrosis when blood flow is obstructed, occurring in approximately 30% of cases. The main cause of vaginal cuff dehiscence with evisceration (VCDE) in young patients is vaginal trauma caused by sexual intercourse, gynecologic instrumentation insertion, or transvaginal ultrasonography/vaginal dilator use before the cuff has completely healed. In elderly patients, evisceration often occurs spontaneously [4,6]. The typical clinical presentation of VCDE is abdominopelvic pain, vaginal bleeding, and fluid discharge, but patients can also present with signs of infection, peritonitis, and very rarely, without symptoms. If VCDE is confirmed, immediate inspection is performed to assess the overall integrity of the bowel [9]. Prolonged bowel ischemia due to delayed management of VCDE can lead to necrosis, which requires a bowel resection and is seen in up to 20% of these patients. VCDE is a life-threatening surgical emergency; therefore, quick recognition and early management are critical [5]. 

Due to its infrequent occurrence, physicians may have little to no exposure to VCDE during training, but the high volume of hysterectomies performed in the U.S. may increase the frequency that a practitioner will encounter VCDE at some point in their career. This lack of exposure during training may delay diagnosis or impede proper management of VCDE, leading to severe, long-term complications or death. Simulation-based training has been found to offer additional benefits to traditional education for all levels of residents, and its use has been widely supported as a pedagogic tool to improve both physician skills and patient outcomes [10,11]. Using simulations for resident training is an effective way to increase symptom recognition and patient safety, as well as provide physicians exposure to rare procedures they do not routinely encounter [12]. The purpose of this simulation is to provide an opportunity for resident physicians to identify VCDE and practice performing a bowel reduction and vaginal cuff repair.

## Materials and methods

Study location and equipment

**Figure 1:**
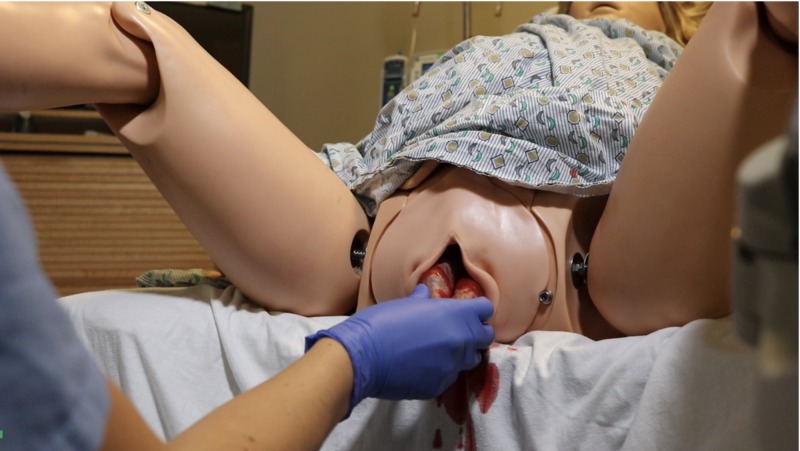
Simulated “bowel” protruding from NOELLE® vaginal introitus

**Figure 2:**
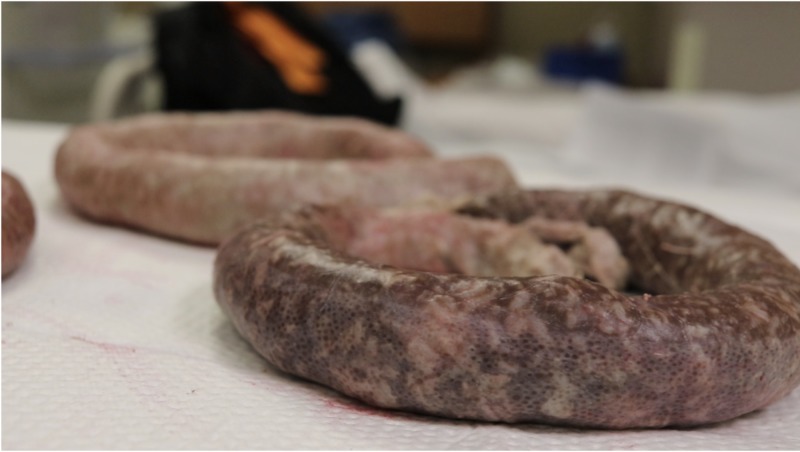
Simulated “bowel” made of chocolate pudding and cooked white rice in sausage casing

The study took place at the Summa Health Akron Campus Virtual Care Simulation Lab in a simulated patient exam room and adjacent simulated operating room. This study was submitted to the institutional review board and qualified for exemption status as an educational intervention. This study utilized a NOELLE® Gynecologic Simulator by Gaumard® (Gaumard Scientific, Florida, US) as a patient named Noelle presenting to the emergency department with VCDE symptoms and simulated “bowel” protruding from the vaginal introitus (Figure 1). A ZOE® Gynecologic Skills Trainer by Gaumard® was modified by creating simulated “bowel” by filling Hog Sausage Casing with two cups of cooked white rice and one chocolate pudding cup, then tying off the casing ends (Figure 2). A vaginal cuff insert was created using Smooth-On Dragon Skin 10 Fast™ Part A and Part B (Smooth-On, Pennsylvania, US). Smooth-On Silc Pig™ flesh coloring was used to make the vaginal cuff model resemble the color of peritoneum. After placing it into the ZOE® gynecologic model, the insert was secured to the model using temporary suture in 4 quadrants, and part of the bowel was then pushed into the open vaginal cuff to simulate evisceration. The ZOE® gynecologic model was set up in the simulated operating room and draped in a fashion consistent with typical surgical practice. The residents were also provided laparotomy sponges, normal saline, a standard laparoscopic instrument tray, and a vaginal surgery instrument tray.

Simulation outline and participants

Thirteen postgraduate year (PGY) 1 to PGY 4 Obstetrics and Gynecology resident physicians volunteered to participate in this simulation. Residents were grouped in pairs of one lower-level resident (PGY 1 or 2) and one upper-level resident (PGY 3 or 4) as would be the case in the typical operating theater. Faculty included a trained Obstetrics and Gynecology physician, a medical simulation expert, and an embedded standardized participant (ESP) serving as a scrub nurse. Each case followed the simulation outlined in Table 1.

**Table 1:** Outline of study design

Pre-Simulation	Simulation	Post-Simulation		
Knowledge Test and Confidence Survey	Simulation - identifying a candidate for surgical management of vaginal cuff dehiscence with bowel evisceration and performing bowel reduction and vaginal cuff closure	Debriefing Session	Didactic Session	Knowledge Test and Confidence Survey

Pre-simulation evaluation and briefing

**Figure 3:**
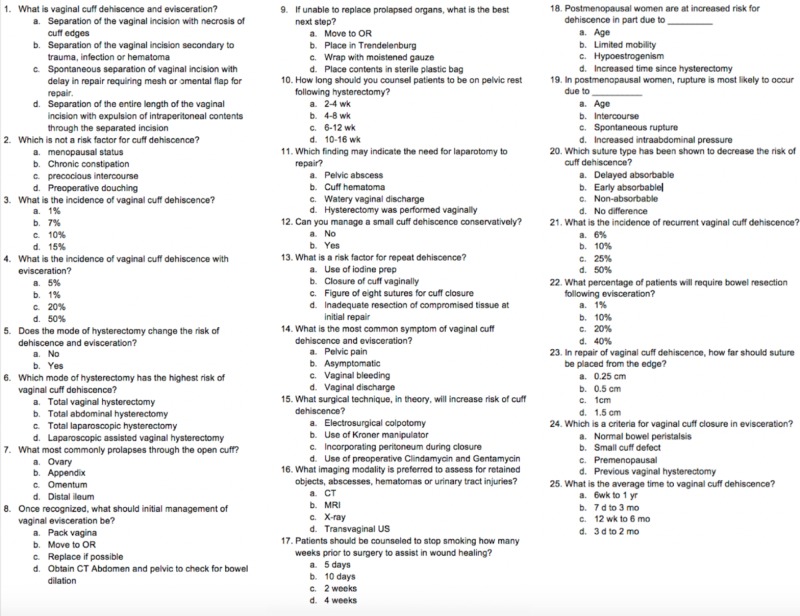
Knowledge test distributed pre- and post-simulation

**Figure 4:**
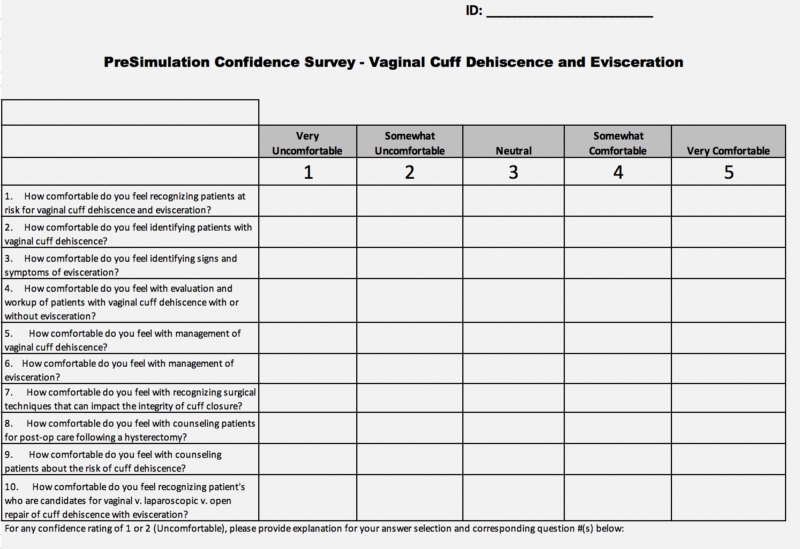
Confidence survey distributed pre- and post-simulation

Before starting the simulation, each resident took a knowledge test and a confidence survey to assess individual understanding and comfort levels of identifying and managing VCDE (Figures 3-4). Residents were then told to treat the simulation as though it were a real-life scenario, and the limitations of the model and simulation environment were explained. Limitations included the rigidity of the pelvic model during the vaginal and speculum exam, lack of active bleeding, and an inability to use electrocautery due to model material. The residents were then given the opportunity to assign roles amongst themselves before starting the simulation.

Simulation case

In the first part of the simulation, the resident pairs were presented with the following case. A 43-year-old female (NOELLE®) presenting to the emergency department for pelvic pressure and vaginal discharge. She is 16 days post-total laparoscopic hysterectomy for abnormal uterine bleeding. She states she was doing fine until the night before when she noticed increased pelvic pressure and vaginal discharge. She also complains of constipation, stating her last bowel movement was four days ago, but denies pain, nausea, vomiting, fevers, chills, and is voiding without difficulty. She is still taking narcotics for post-operative pain management, but is not on a stool softener.

Her vitals upon arrival (VS 1) are a blood pressure of 136/72, heart rate of 90, respiratory rate of 16, and a temperature of 37.2C. The physical exam reveals a regular heart rate and rhythm with lungs clear to auscultation bilaterally. Her abdomen is soft, non-distended, and not-tender with decreased bowel sounds. Pelvic exam reveals watery vaginal discharge and a pink mass, “bowel,” protruding from the vaginal introitus.

**Figure 5:**
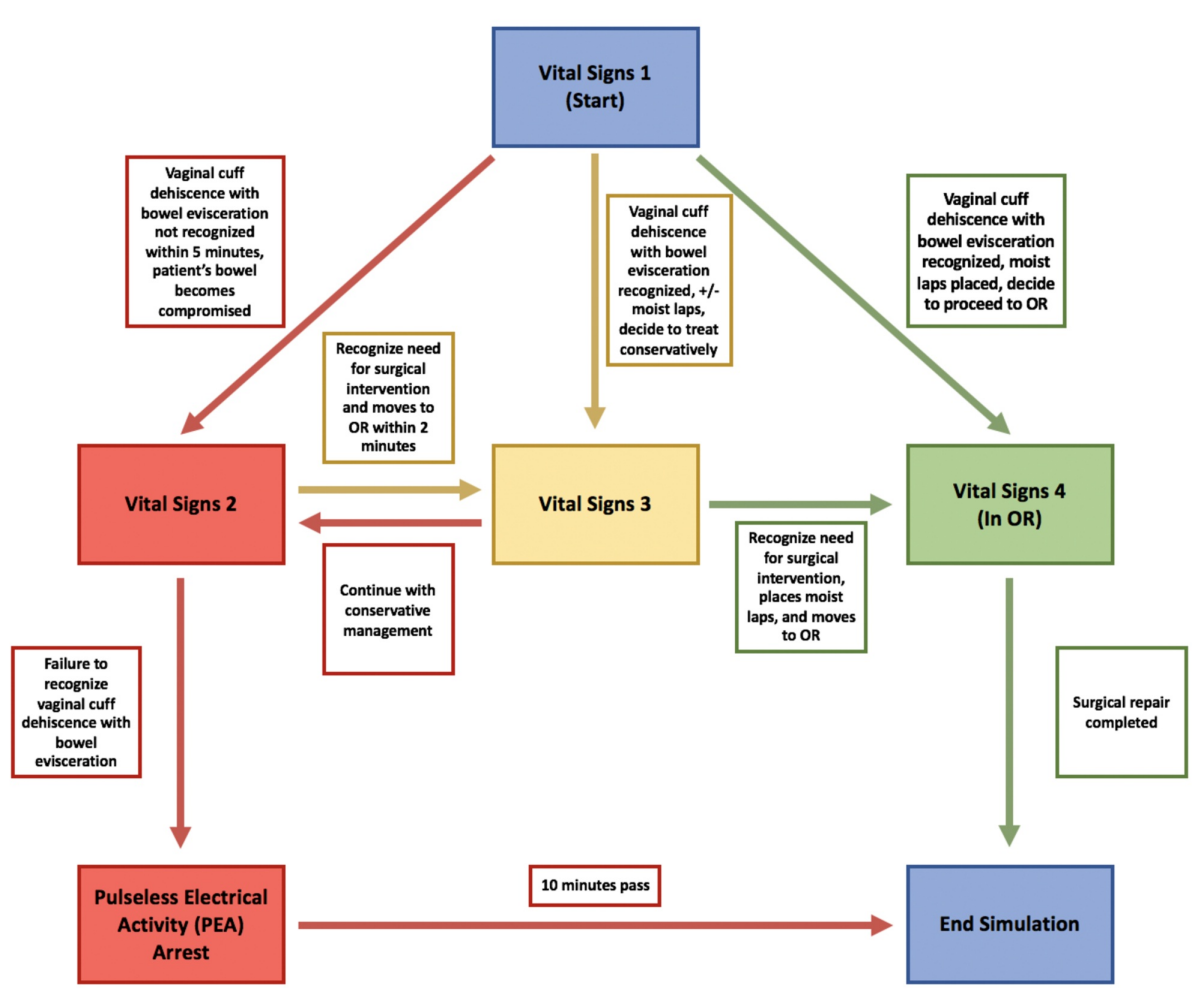
Flow chart of patient condition as simulation progressed based on resident physician management decisions

The residents were able to request a complete metabolic panel and a complete blood count that were representative of a patient presenting at the hospital with the above complaints. NOELLE’s® vital signs as seen in Table 2 and labs worsened if the residents failed to identify and intervene appropriately (Figure 5).

**Table 2:** Vital signs for simulation

Vital Signs (VS)	Hearts Rate (beats/min)	Blood Pressure (mmHg)	Temperature (C)	O2 Saturation	Respiratory Rate (breaths/min)
VS 1	90	136/72	37.2	98%	16
VS 2	130	85/56	39	94%	22
VS 3	120	94/58	38.2	96%	18
VS 4	92	128/71	37.4	98%	12

**Figure 6:**
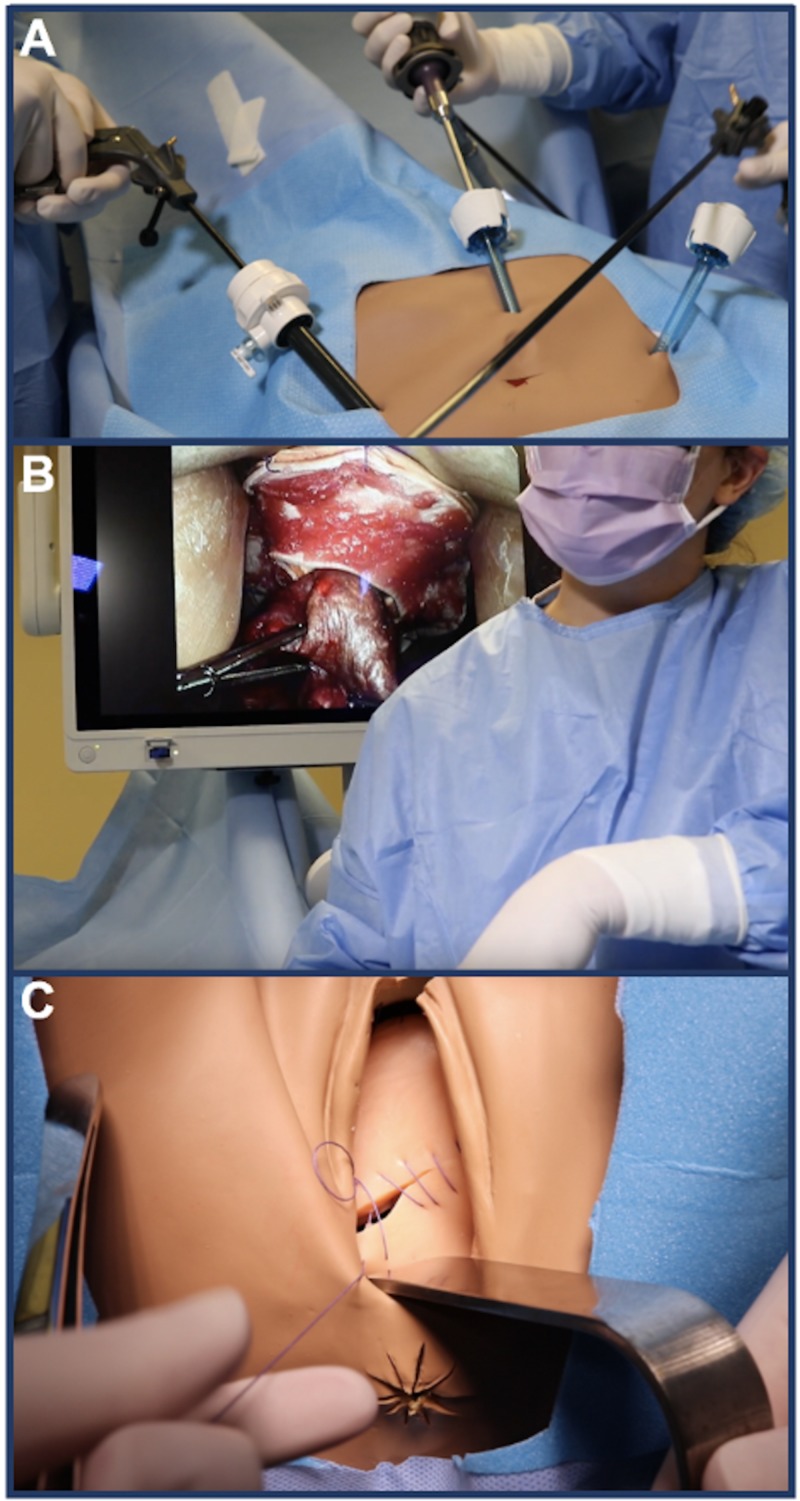
ZOE® gynecologic model with laparoscopic ports draped (A) for bowel evisceration reduction (B) and subsequent vaginal cuff closure (C)

Once the VCDE was recognized and surgical intervention was determined, participating residents made the decision to transfer NOELLE® to the adjacent operating room. In the adjacent operating room, the modified ZOE® was prepped and draped to perform a reduction of the bowel evisceration, as seen in Figures 6A-6B, and a vaginal cuff closure either laparoscopically or vaginally based off of surgeon preference (Figure 6C). The simulation ended once the surgical repair was completed.

Post-simulation evaluation and debriefing

Following the simulation, debriefing and didactic sessions occurred to allow residents time to discuss their thoughts on how they managed the case. This helped them reflect and identify any errors in their management, discuss case-specific findings and treatment, and allow for reinforcement of improvement strategies for future cases. After completing the didactic session, each resident repeated the knowledge test and confidence survey to identify knowledge deficits that persisted after the experience. The goal of the post-simulation evaluation was to identify critical points of training that need to be re-emphasized, as well as assess the educational curriculum. A Wilcoxon signed rank test was performed to test whether the median change in scores is equal to zero, and statistical significance, p<0.05, was evaluated for the knowledge test and confidence survey.

## Results

**Figure 7:**
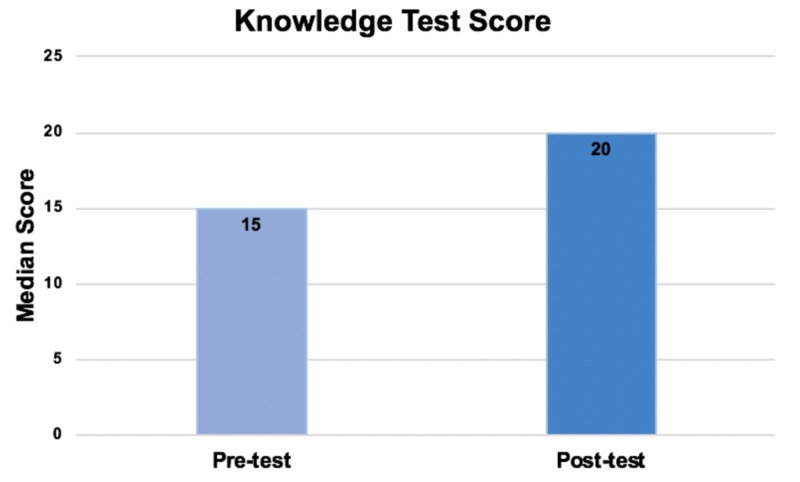
Knowledge test scores

**Figure 8:**
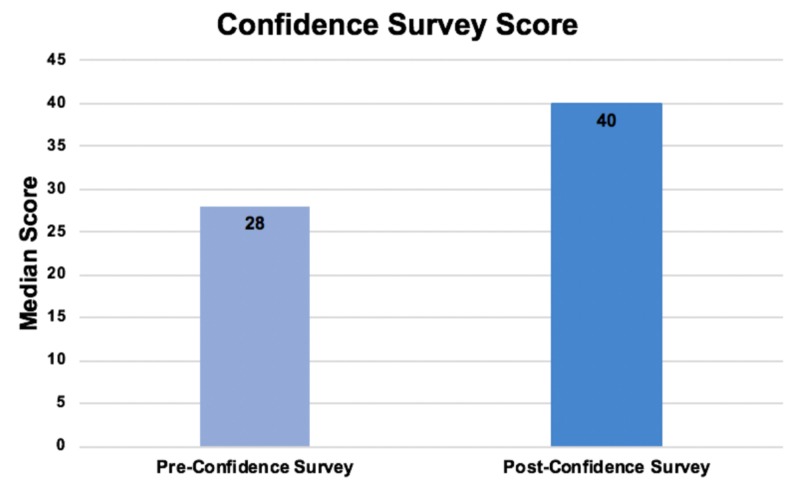
Confidence survey scores

The knowledge test, which had a maximum score of 25, had a pre-test median score (and interquartile range(IQR)) of 15(12-18). The post-test had a median score (and IQR) of 20(19-22), giving a median score change (and IQR) of 5(3.5-8.5) (p=0.001) (Figure 7). The confidence survey had a maximum score of 50, and median (and IQR) pre- and post-confidence survey scores of 28(20-34.5) and 40(37.5-46) respectively, giving a median score change (and IQR) of 15(8-20.5) (p=0.001) (Figure 8).

Kirkpatrick level 1 was assessed through the confidence survey and the post-simulation debriefing session. Learners expressed appreciation for the relevance of the simulation as well as the opportunity to practice repair of a VCDE. Kirkpatrick level 2 was assessed through the pre- and post-simulation knowledge test and confidence survey. These scores were analyzed to determine how much the participants comprehended and how their comfort levels changed following the intervention.

## Discussion

Our results demonstrate a statistically significant increase in residents’ knowledge and confidence in recognizing and managing VCDE after participation in this curriculum. While it is rare, it is important for residents to recognize presenting symptoms of VCDE and demonstrate effective management procedures, as it is a surgical emergency they will likely encounter with many associated morbidities. Management and repair of VCDE is also associated with risks, as 15% of patients will experience more postoperative complications, and there is a 6% recurrence rate of cuff dehiscence [6]. With continuous practice and education, patient care will be improved, and as residents become stronger at recognizing and treating VCDE, they can further improve on their bowel reduction techniques and vaginal cuff repairs, potentially leading to reduced postoperative complications and recurrence [12].

Some strengths of the study include its relevance to Obstetrics and Gynecology physicians and its reproducibility, making it feasible to train residents on rare conditions. Not only does the use of simulation provide exposure to less frequently performed procedures, it actively engages participants in the learning process, providing deliberate practice in a well-controlled, low-risk environment.

Limitations of the study include a small sample size and a narrow timeframe. Further, reproducing the study at a different location would require that location to have equipment that can be modified to function with the case scenario. The pre- and post-simulation questionnaires and surveys were done on the same day, so the curricular impact long term cannot be assessed. Lastly, it is important to discuss the nature of running a simulation as a limitation in and of itself. While simulations present a great opportunity to teach residents, the events unfold in an expedited time frame and there are limitations on how realistically one can present a case.

Although this intervention only measured the impact of Kirkpatrick levels 1 and 2 in regard to residents' feelings and knowledge of VCDE, future studies aimed at evaluating Kirkpatrick levels 3 and 4 can be implemented by reviewing patient outcomes following training with this curriculum. Furthermore, expanding the simulation beyond one institution to increase the number of participants could benefit a greater number of residents. Finally, involving various interprofessional teams in the simulation, such as nurses or operating room staff members instead of embedded standardized participants, could help teach early recognition and management to other providers besides the physician and improve overall patient care.

## Conclusions

The intervention taught residents how to recognize patients presenting with VCDE, appropriately manage them, and perform a reduction of prolapsed bowel and vaginal cuff repair. Simulation-based training can lead to improved behaviors of learners, which can translate into decreased procedural errors and improved patient safety. This study suggests using simulations is an effective way to improve resident physicians’ knowledge and confidence regarding rare, life-threatening emergencies they are not typically exposed to during training, such as VCDE.
